# A Case of Fatal Catatonia in a COVID-19 Patient

**DOI:** 10.7759/cureus.16529

**Published:** 2021-07-21

**Authors:** Hyuck-Jin Kwon, Krunal H Patel, Miguel Ramirez, Isabel M McFarlane

**Affiliations:** 1 Internal Medicine, State University of New York (SUNY) Downstate College of Medicine, Brooklyn, USA; 2 Internal Medicine, State University of New York (SUNY) Downstate Medical Center, Brooklyn, USA

**Keywords:** fatal catatonia, covid 19, bush francis catatonia scale, catatonia, covid-19 cns involvement

## Abstract

COVID-19 has been associated with numerous complications, primarily pulmonary in origin. However, there have been several neurological sequelae of COVID-19 as well, one of the rarer complications is catatonia. In this already vulnerable population, it is imperative for the early diagnosis of catatonia and starting treatment. Delay in treatment of catatonia can be fatal from secondary complications as seen here. We discuss a case of a 62-year-old female that presented with mild COVID pneumonia, subsequently developed catatonia precipitated by COVID-19 encephalitis, which ultimately led to her death from complications.

## Introduction

Catatonia can be observed in patients with an active viral infection with central nervous system (CNS) involvement [1]. There is a precedent of viral infections precipitating a catatonic state. In the immune model of catatonia, there have been historical citations of catatonia induced by neuroinvasion by the infectious agent [2]. This type of postinfectious catatonia was first recognized in 1916 and became widespread with the influenza pandemic of 1918 it was labeled as an amyostatic-akinetic form known as encephalitis lethargica [2]. During this time, scientists sought to understand the neurobiological etiology of the disease. It was discovered that in patients presenting with psychiatric symptoms, there was marked atrophy of the basal ganglia, midbrain, and hypothalamus, pointing to a pathophysiological etiology to the psychiatric symptoms seen in patients with encephalitis lethargica after their influenza infection [3].

With the COVID-19 pandemic, there have been reports in which SARS-CoV-2 has been found in the cerebrospinal fluid (CSF) of COVID-19 patients based on polymerase chain reaction testing for the virus [4]. Other reports have found that patients present with a catatonic state due to encephalopathy secondary to an aberrantly excessive innate immune response [4]. Due to the immune response in these patients, there have been reports of global brain dysfunction and severe psychomotor retardation [4]. In these cases there has been an absence of CSF abnormalities, suggesting even in the absence of CSF findings, inflammatory damage by SAR-CoV-2 cannot be ruled out [4]. The current theory is that there are innate immune responses in the CNS that are signaled due to the surges of pro-inflammatory cytokines in the peripheral immune system, which in turn will disrupt the blood-brain barrier [4]. Additionally, there is likely an association with the angiotensin-converting enzyme 2 (ACE-2) which has been found not only in respiratory and vascular endothelium but also in brain tissue [5].

Catatonia historically is underdiagnosed and undertreated; therefore, it is easy to miss the signs of catatonia in patients who are acutely ill or develop a myriad of complications such as dehydration, malnutrition, aspiration, pneumonia, pressure ulcers, and thromboembolism [4] Catatonia is a life-threatening medical condition which if untreated can result in death. Many patients with catatonia go on to become dehydrated and immobile, which can precipitate a thromboembolic event such as a pulmonary embolism as we will describe here. It is essential to identify and diagnose patients urgently as prolonging time to intervention is associated with worse morbidity and mortality. Given that catatonia has a definitive treatment and responds to benzodiazepines, it is a diagnosis that should not go unmissed in this already vulnerable population.

## Case presentation

A 62-year-old woman with a past medical history of hypertension, schizoaffective disorder, and bipolar disorder presented to the emergency department with shortness of breath and weakness for one week. The patient was found to be COVID-19 positive. The patient denied fever, chills, nausea, vomiting, and diarrhea. The patient’s vitals at the time were T 98.8, BP 150/71, HR 89, RR 20, Percent oxygen saturation 89% on room air. While on a high-flow nasal cannula (FiO_2_ 90% 30 LPM) she was saturating above 94%. The patient was on the following home medications: amlodipine 5mg, hydrochlorothiazide 25mg, losartan 100mg, and quetiapine 300mg.

The patient was alert and oriented x4 on presentation, with normal mood and affect. There were no acute events during hospital days 2-4, but on hospital day 5, the patient was observed to be speaking in Haitian Creole, when the team had been communicating with the patient in English prior. After using translation services, the patient refused to speak with the doctor or the interpreter.

During hospital days 6-9, the patient displayed extreme mutism and negativism, not speaking or responding to any commands. On hospital day 9, a Creole-speaking nurse was brought to the room, and attempted to video call her brother; she was unresponsive to her brother as well. CT scan of the head was negative with no acute findings, and EEG showed moderate diffuse cerebral dysfunction, non-specific in etiology. Triphasic waves, classically associated with toxic metabolic dysfunction were seen, pointing to encephalitis likely from COVID-19. Lumbar puncture was discussed however her brother opted to hold off. On hospital day 10, the patient showed continued altered mental status and received a single 1mg trial of Ativan (lorazepam) for a presumed diagnosis of catatonia with no improvement. By hospital day 12, the patient was nonverbal. On the physical exam, her lower extremities were stiff; however, she was able to straighten her legs passively with difficulty, demonstrating a wax-like movement. Upper extremities were also stiff, moving with passive movement only, and would remain in a locked wax-like position. On hospital day 13, a brain MRI was performed and was also negative of acute or chronic intracranial pathology. Her Bush Francis Catatonia score was 13, indicating a catatonic state. 

On hospital day 14, the patient's results came back COVID negative, mental status was unchanged from days 14-17. On hospital day 18 from the recommendations of the psychiatric team, the patient was started on an Ativan challenge withstanding 1mg Ativan every eight hours intravenously. On hospital day 19, the patient was seen to be much more aware of her surroundings and was more verbal. Her movements were still slow and there were moments where the patient receded into a catatonic state. On hospital day 20, after 48 hours of starting the Ativan challenge, the patient had a significant improvement in her clinical state. Bush Francis' score repeated was 6, which was decreased from 13. The patient continued to show improvement and was able to respond to questions, and was now alert and oriented x3. There was still some residual rigidity and occasional regression; however, with the standing Ativan added, there was a significant improvement in her catatonic state as demonstrated by the Bush Francis score.

**Figure 1:**
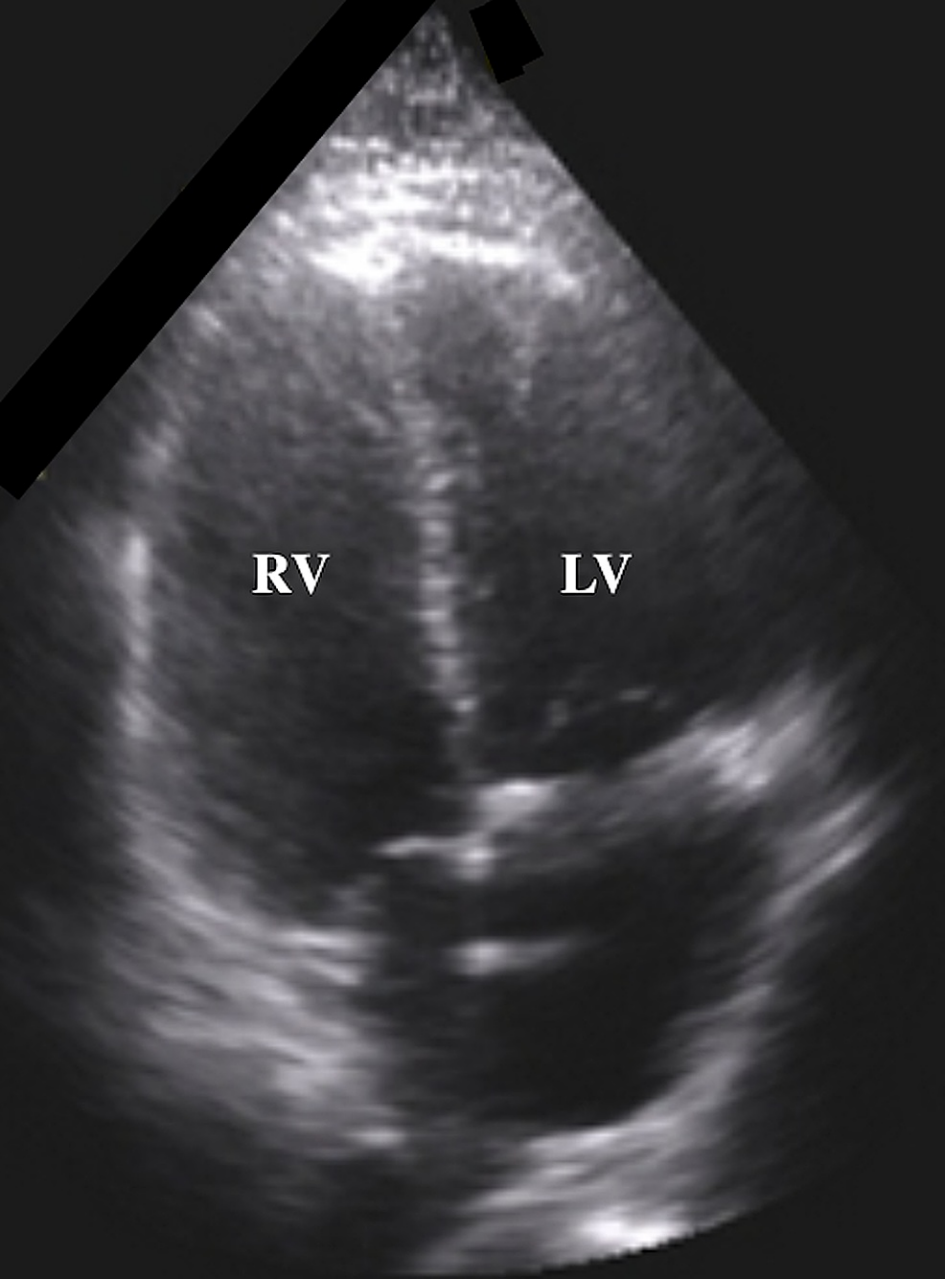
Bedside point of care ultrasound (POCUS) during CPR showed a dilated RV and an RV:LV ratio greater than 1, indicating an increase in RV afterload likely secondary to a PE given the clinical context. CPR - cardiopulmonary resuscitation; RV - right ventricle; LV - left ventricle

During the afternoon of hospital day 20, a rapid response was initiated which later turned into a cardiac arrest code once no pulses were palpated at the bedside. During the cardiopulmonary resuscitation (CPR) efforts a point of care ultrasound (POCUS) examination was done of the heart. POCUS of the heart showed a dilated right ventricle (RV) (Figure 1) compared to the left ventricle (LV).

The RV to LV ratio was estimated to be greater than 1. Additionally, septal bowing into the LV was seen on POCUS in the parasternal short-axis view, indicating a “D-sign,” not pictured here. No pericardial effusion was seen. Given these findings, it was suspected the sudden cardiac arrest refractory to resuscitation efforts was likely secondary to a massive pulmonary embolism. After several rounds of CPR, the patient was pronounced deceased.

## Discussion

Currently, there are few reported cases of SARS-CoV-2 infection and catatonia. In the past year, there have been numerous cases of SARS-CoV-2 infections, and while neuropsychiatric symptoms are very rare, catatonia is one of the sequelae that can be diagnosed and treated with improvement in a short period of time. According to DSM V criteria, catatonia is diagnosed when three or greater symptoms are present. The symptoms as per the DSM V criteria are stupor, catalepsy, waxy flexibility, mutism, negativism, posturing, mannerism, stereotypy, agitation, grimacing, echolalia, and echopraxia [6]. In the case of our patient, there was a display of stupor, mutism, negativism, grimacing, and catalepsy [6,7]. Additionally, the Bush Francis catatonia rating scale has a sensitivity close to 100% for the diagnosis of catatonia [6]. Our patient scored 13 on the scale, an improvement of 50% or more after standing Ativan is also specific for catatonia. The patient did not display any of these symptoms on admission, and they presented later on in the course of her hospitalization. This may have been due to the fact that her SARS-CoV-2 viral load may have become significantly higher later in her hospitalization or from the secondary inflammatory response causing her to have symptoms with progressive CNS involvement [8].

After observing the signs of catatonia for several days without improvement, a standing Ativan challenge was implemented as per the recommendations of the psychiatric team. The reason the Ativan was not given until later in the hospitalization was because of the challenges of managing other medical problems with patients with SARS-CoV-2 infections. Patients with COVID-19 are at risk of developing hypoxemic respiratory failure and this was a potential risk of standing Ativan as it is a respiratory drive depressant [9]. The day after the Ativan challenge was conducted, the patient had significant improvement in her mental status. The patient was able to have a fluent conversation in English again and was noted to be alert and oriented to time, person, and place. She was starting to ask questions regarding her hospital stay which was also an improvement, engaging with the hospital staff appropriately. Her physical examination was also noted to have improved. She was no longer rigid or in a stupor, she was observed to be eating on her own without assistance. She was also then able to move all upper and lower extremities upon request and spontaneously. Due to the marked improvement, after Ativan was administered, it was clear that the patient had sequelae of catatonia that began during her hospitalization, most likely triggered by her SARS-CoV-2 infection [10].

Once she was placed on the proper treatment regimen she had a significant improvement in her oral intake as well as her overall mobility. Her pulmonary embolism, as per Virchow's triad, was likely a complication of venous stasis and hemoconcentration causing hypercoagulability secondary to immobility and dehydration from her catatonic state [11]. Of note, COVID-19 is associated with the thromboembolic disease; however, by hospital day 20, our patient did not have active sequelae of COVID-19 at this time. It is difficult to say without a doubt, but if proper diagnosis and treatment had been initiated earlier it is possible her terminal event of the pulmonary embolism may have been avoided. Decreased mobility and hemoconcentration likely precipitated a deep vein thrombosis which likely resulted in a PE. This cannot be confirmed with 100% certainty as the autopsy was deferred but can be inferred based on the sudden deterioration in clinical status and echocardiography.

Since the start of the pandemic, COVID-19 has caused a great deal of morbidity ranging from pulmonary and circulatory compromises to lesser-known sequelae such as catatonia described here or its bizarre interactions with medications [12].

## Conclusions

Given that the patient did not have active sequelae of her COVID-19 during her death, it is postulated that her massive pulmonary embolism was likely a complication of immobility coupled with decreased oral hydration from her catatonic state. It is imperative that clinicians be aware of this fatal neurological complication of COVID-19, as early treatment is key. Prevention of venous thromboembolism by reducing the risk factors and relieving catatonic symptoms early is essential in the management of this readily treatable condition in this vulnerable population.
